# Directional ballistic transport in the two-dimensional metal PdCoO_2_

**DOI:** 10.1038/s41567-022-01570-7

**Published:** 2022-05-09

**Authors:** Maja D. Bachmann, Aaron L. Sharpe, Graham Baker, Arthur W. Barnard, Carsten Putzke, Thomas Scaffidi, Nabhanila Nandi, Philippa H. McGuinness, Elina Zhakina, Michal Moravec, Seunghyun Khim, Markus König, David Goldhaber-Gordon, Douglas A. Bonn, Andrew P. Mackenzie, Philip J. W. Moll

**Affiliations:** 1 Max Planck Institute for Chemical Physics of Solids, Dresden, Germany; 2 School of Physics and Astronomy, University of St Andrews, St Andrews, UK; 3 Department of Applied Physics, Stanford University, Stanford, CA USA; 4 SLAC National Accelerator Laboratory, Menlo Park, CA USA; 5 Department of Physics and Astronomy & Stewart Blusson Quantum Matter Institute, University of British Columbia, Vancouver, British Columbia Canada; 6 Department of Physics, Stanford University, Stanford, CA USA; 7 Institute of Materials, École Polytechnique Fédérale de Lausanne (EPFL), Lausanne, Switzerland; 8 Department of Physics, University of Toronto, Toronto, Ontario Canada

**Keywords:** Electronic properties and materials, Electronic properties and materials

## Abstract

In an idealized infinite crystal, the material properties are constrained by the symmetries of the unit cell. The point-group symmetry is broken by the sample shape of any finite crystal, but this is commonly unobservable in macroscopic metals. To sense the shape-induced symmetry lowering in such metals, long-lived bulk states originating from an anisotropic Fermi surface are needed. Here we show how a strongly facetted Fermi surface and the long quasiparticle mean free path present in microstructures of PdCoO_2_ yield an in-plane resistivity anisotropy that is forbidden by symmetry on an infinite hexagonal lattice. We fabricate bar-shaped transport devices narrower than the mean free path from single crystals using focused ion beam milling, such that the ballistic charge carriers at low temperatures frequently collide with both of the side walls that define the channel. Two symmetry-forbidden transport signatures appear: the in-plane resistivity anisotropy exceeds a factor of 2, and a transverse voltage appears in zero magnetic field. Using ballistic Monte Carlo simulations and a numerical solution of the Boltzmann equation, we identify the orientation of the narrow channel as the source of symmetry breaking.

## Abstract (web summary)

Electrons in PdCoO_2_ can travel a long way before being scattered, and their band structure is such that they can travel in only one of three directions. As a result, the current flow through this nanoscale conductor can be very efficient.

## Main

The possibility of directionality, or anisotropy, of the electrical resistivity of a crystalline material is determined by the point-group symmetry of its underlying lattice. The resistivity *ρ* in two dimensions for square, triangular and hexagonal lattices must be isotropic (Supplementary Note 1). Only if the rotational symmetry is lowered to two-fold is in-plane anisotropy permitted, such that the two diagonal components *ρ*_*xx*_ and *ρ*_*yy*_ differ. Such symmetry lowering has attracted significant attention due to the study of so-called electronic nematic and smectic liquid crystals, in which self-organization of the electron fluid is thought to be the driver of the broken symmetry^1–5^. Indeed, transport measurements sensitive to resistive anisotropy have been a key probe of this class of physics. In this work, we address the question of whether other approaches can also induce transport anisotropies. Specifically, we create micron-scale devices of differing orientation relative to an underlying crystal lattice, without disturbing the point-group symmetries of that lattice, and investigate whether channel orientation affects transport.

Fundamentally, all bulk crystalline symmetries are broken in any finite-size conductor. However, this usually has no observable effects on measured resistances of metals because carriers scatter so strongly in the bulk that scattering at the boundaries is irrelevant. In this case, the current density and electric field are related by a resistivity tensor *ρ* that adheres to the crystalline point-group symmetry. It has been known for decades that it is possible to purify metallic and semiconducting crystals enough to enter the so-called ballistic transport regime^6,7^, in which the electron mean free path *λ* between internal scattering events exceeds the minimum sample dimension. In this regime, boundary scattering becomes relevant or even dominant. However, this alone is not sufficient to produce observable resistivity anisotropy. The essential additional ingredient is significant Fermi surface (FS) anisotropy. Early theoretical consideration of ellipsoidal Fermi surfaces^8–11^ led to predictions of transport anisotropies, but experiments in aluminium^12,13^ did not resolve such effects. Recent results on epitaxial tungsten thin films have detected a growth-direction dependence of the resistance when the films are thin enough to be in the ballistic limit in the direction perpendicular to the substrate^14^. This result is attributed to the anisotropy of the three-dimensional Fermi surface of tungsten and hence suggests that boundary-induced symmetry breaking is achievable. Here, we exploit the in-plane anisotropy of the Fermi surface in a two-dimensional metal, PdCoO_2_, to demonstrate not only that directional symmetry breaking is achievable but that it can be a large effect. By cutting differently oriented channels from the same single crystal, we remove any sample-dependent uncertainties from the experiments. We present a simple intuitive picture to explain our observations, and then reinforce it with calculations and Monte Carlo simulations.

## Experiment

The ultra-clean, naturally layered crystal structure of PdCoO_2_ is host to extremely conductive, quasi-two-dimensional sheets of palladium, separated by layers of CoO_2_ octahedra. Due to the strikingly high purity of this oxide^15^, it can support electron mean free paths of up to 20 µm at temperatures below 20 K, as evidenced by an in-plane residual resistivity value of only 8 nΩ cm (ref. ^16^). Extensive de Haas–van Alphen^17^, angle-resolved photoemission^18,19^, angle-dependent magnetoresistance^20^ and magneto-transport^21^ measurements have well characterized its hexagonal Fermi surface, which fills half of the Brillouin zone, as expected for a monovalent metal. The out-of-plane dispersion is so weak that transport is essentially two dimensional (2D)^22^, permitting to work in a 2D approximation where the material is considered to be a stack of independent 2D layers conducting in parallel. The extremely long in-plane mean free path of PdCoO_2_ has been demonstrated directly in measurements of transverse electron focusing^23^ and the observation of field-periodic oscillations in microstructures^24^.

**Fig. 1:**
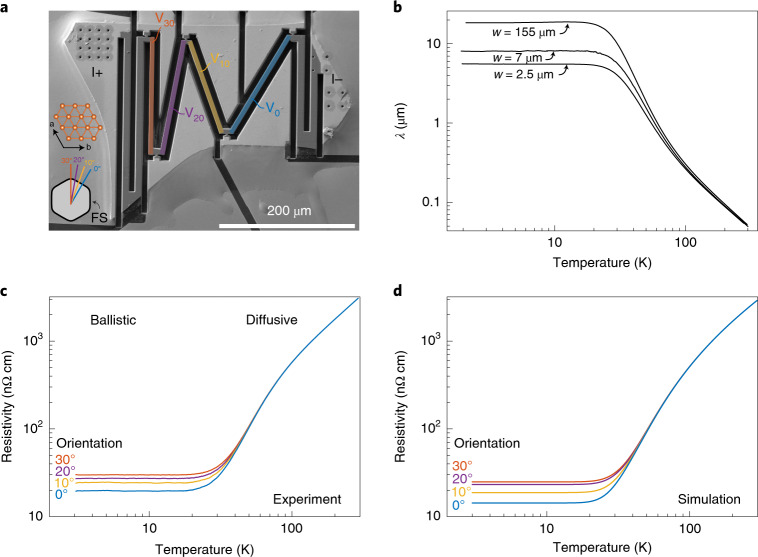
Temperature-dependent in-plane transport of PdCoO_2_. **a**, One of the PdCoO_2_ single-crystal devices used in this work. To ensure a homogeneous current flow throughout the full thickness of the crystal, despite the large resistivity anisotropy of over $\rho _c/\rho _a$ > 2,000 below 20 K, a long current-injection meander between the current terminals I+ and I- has been carved into the device layout^25^. In this way, the resistivity can be accurately determined along the four subsequent transport bars (V_0_, V_10_, V_20_ and V_30_) simultaneously. Five voltage contacts are distributed along the turning points of the zigzag current path. Additionally, the real-space orientation of the Fermi surface with respect to the crystal is shown. The apparent resistivity of each bar is defined as the measured voltage divided by the sourced current multiplied by the appropriate geometrical factor. When the sample is in the diffusive regime this corresponds to the bulk resistivity of PdCoO_2_, but when the ballistic regime is entered it becomes a device-specific quantity. The transport bars of the depicted device have a uniform thickness of 7.8 µm, and a width *w* and length *l* of *w*_0_ = 7.3 µm and *l*_0_ = 170.6 µm for V_0_, *w*_10_ = 6.5 µm and *l*_10_ = 146.5 µm for V_10_, *w*_20_ = 7.4 µm and *l*_20_ = 172.5 µm for V_20_ and *w*_30_ = 6.7 µm and *l*_30_ = 174 µm for V_30_. **b**, The temperature-dependent mean free path *λ* of a PdCoO_2_ bulk sample (155 µm wide; data replotted from ref. ^16^) and those from the 30° bar pictured in **a** at its initial width of 7 µm and after narrowing to a width of 2.5 µm, all calculated using a standard 2D expression (Supplementary Note 3). **c**, The temperature-dependent resistivity of the four transport bars shown in **a**. In the diffusive regime, all four curves collapse onto the same value, whereas the transport is governed by ballistic effects at lower temperatures. As a result, the residual resistivity is enhanced compared with the bulk value (8 nΩ cm (ref. ^16^)) due to boundary scattering. Strikingly, this resistivity enhancement is strongly angle dependent. **d**, Results from Boltzmann transport simulations taking into account the realistic Fermi surface shape as well as the temperature-dependent bulk mean free path (Supplementary Note 5). Source data

The crystals of PdCoO_2_ used in this study grow as thin platelets with a typical thickness of approximately 5–30 µm and lateral dimensions of several hundred micrometres. Despite their layered structure, the crystals cannot be exfoliated and laid on a separate substrate to be patterned and contacted by using conventional nanofabrication techniques. Instead, we employ a focused ion beam (FIB) for three-dimensional microsculpting of as-grown crystals. As shown in Fig. 1a, a crystal platelet, about 350 µm long and 8 µm thick, has been anchored to a sapphire substrate using two-component Araldite epoxy. A thin layer of titanium/gold (10 nm/150 nm) has then been evaporated(Ti)/sputtered(Au) on top of the device to create electrical contacts to the crystal. In a final step, first the titanium/gold layer is locally removed by FIB etching and subsequently a transport bar is shaped into the crystal with suitable voltage contacts lengthways. The FIB-induced surface damage is limited to the outermost approximately 20 nm (Supplementary Note 2) and does not lead to any bulk defects^25,26^, as the ballistic transport observed in the FIB-cut bars self-evidences. Details of crystal synthesis^23^ and FIB microstructuring^23,26^ are given elsewhere. A typical PdCoO_2_ transport device produced by FIB micromachining is displayed in Fig. 1a. Conveniently, the growth edges of the PdCoO_2_ crystals are oriented perpendicular to the crystallographic axes, so that the crystal orientation can be determined easily. This permits the fabrication of four serial transport bars precisely oriented with respect to the crystal lattice of the same single crystal. Because of the in-plane six-fold rotational symmetry and reflection symmetry of the palladium planes, the full angular range can be spanned in steps of 10° by choosing to measure parallel to the crystal direction ([110], here denoted ‘0°’) as well as 10°, 20° and 30° away from the [110] direction. Further, we note that throughout this manuscript we present our data in terms of resistivities, defined by the measured voltages divided by the applied constant current.

## Results

### Directional ballistic effects

The in-plane mean free path *λ* can be estimated from the resistivity under the assumption of 2D transport as $\rho ^{ - 1} = \frac{{e^2}}{{hd}}k_{\mathrm{F}}\lambda$, where *d* = 17.73/3 Å is the palladium layer separation and *k*_F_ = 0.95 Å^−1^, where *k*_F_ is the average Fermi wavevector around the Fermi surface (see Supplementary Note 3 for details). Figure 1 contrasts *λ* of the 0° oriented bar as a function of temperature to that of a bulk (155 µm wide) channel of the same orientation. Data are also shown for the same bar after subsequent narrowing from 7 to 2.5 µm width and are seen to evolve in a text-book fashion: At high temperatures, λ is strongly limited by phonon scattering and the sample is in a diffusive transport regime, hence its electronic response is width independent. At low temperatures, this is no longer the case and the low-temperature value of λ for the restricted channels is limited by their width rather than by bulk scattering. The temperature at which the data first deviate from the diffusive regime is therefore itself width dependent (see Supplementary Note 3 for details).

Figure 1c shows the angular dependence of the in-plane resistivity as a function of temperature. While the resistivity is isotropic above about 50 K, as is expected in the diffusive transport regime, it becomes remarkably anisotropic at lower temperatures, where the electron mean free path exceeds the width of the transport bars. In particular, for the device displayed in Fig. 1a in which the bars are 7 µm wide, the resistivity anisotropy $(\rho _{30} - \rho _0)/\rho _0$ is as large as 50% between the most and least resistive direction. Upon thinning down the bars to 2.5 µm width, this ratio further increases to 200%.

**Fig. 2:**
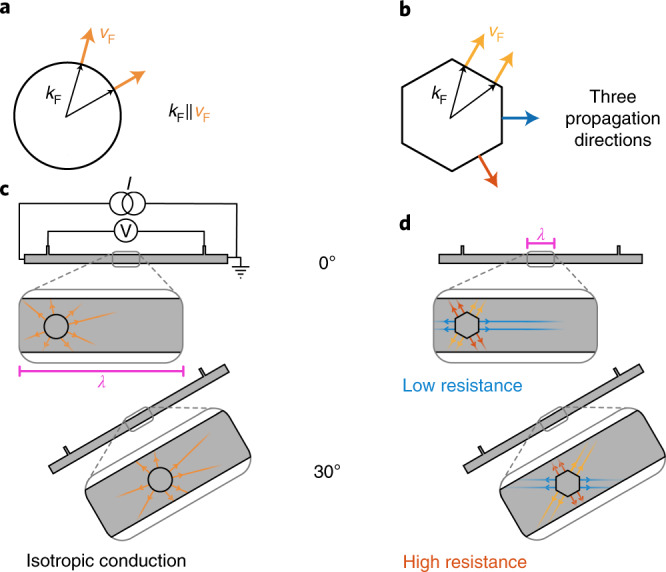
Ballistic electron propagation in the case of a circular and a hexagonal Fermi surface. **a**, The direction of the Fermi velocity *v*_F_ is always parallel to the Fermi momentum *k*_F_ for a circular Fermi surface. **b**, The situation is drastically different in the case of a hexagonal Fermi surface. Due to the flat sides of the polygon, there are only three possible directions for the Fermi velocities (yellow, blue and red). This restriction of the electron propagation direction results in highly anisotropic, directional ballistic transport. **c**, The electronic conduction in a four-point transport bar fabricated from a material with an isotropic Fermi surface will not depend on the orientation of the bar. **d**, In contrast, for a hexagonal Fermi surface, a bar cut parallel to an electron propagation direction will show a lower resistance than a bar aligned perpendicular to an electron propagation direction.

The order of the curves in Fig. 1c, from least to most conductive, can be understood qualitatively by considering Fig. 2. When the transport bar is oriented such that it is aligned with one of three main directions of the Fermi velocity, a large number of electronic states propagate parallel to the bar and avoid any surface collisions. On the other hand, when the orientation of the transport bar is rotated by 30°, the dominant ballistic directions guide the electrons towards the sample edges, leading to frequent boundary scattering events.

**Fig. 3:**
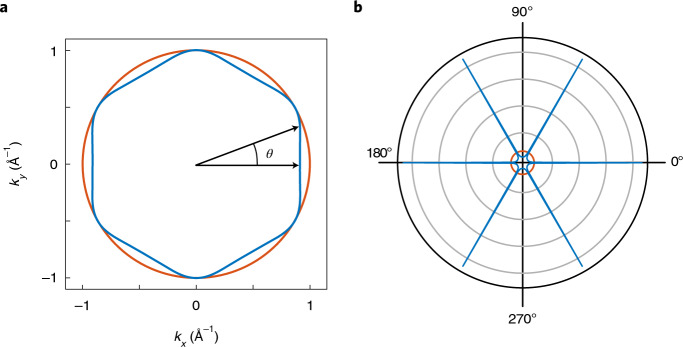
The Fermi velocity density and directional ballistics. **a**, The Fermi surface of PdCoO_2_ (blue) compared with a circle (red). *θ* denotes the angle from the [110]-direction. At first sight the difference between the two is small. **b**, In contrast, the Fermi velocity direction distributions of the two Fermi surfaces are vastly different, with this difference leading to the strong directional ballistic effects that we have measured. While an isotropic Fermi surface (red) leads to a corresponding isotropic Fermi velocity direction distribution, the faceted Fermi surface of PdCoO_2_ (blue) results in a Fermi velocity direction distribution which is sharply peaked along six directions. Note that the velocity direction distribution has been normalized the same for both Fermi surfaces. The sharp spikes in **b** emphasize that each facet of the PdCoO_2_ Fermi surface contains a large number of states with the same direction of Fermi velocity.

These results clearly demonstrate the notion of directional ballistics. In any material with a circular Fermi surface, the apparent resistivity is enhanced due to boundary scattering, depending on the specularity of the boundaries^27^. This effect is isotropic: a bar of a given width and length will have the same resistance no matter what orientation it is cut in. Most high-mobility two-dimensional electron gases have circular or smoothly evolving Fermi surfaces, in which no orientation dependence is observable. In contrast, a Fermi surface with a strongly non-isotropic Fermi velocity distribution can significantly modify the rate of boundary scattering and therefore support an orientation dependence of the resistance. A comparison of the Fermi surface shape and the velocity density map highlights the subtle role of anisotropy (Fig. 3). The overall 2D Fermi surface of PdCoO_2_ does not deviate much from a circular approximation, and given its six-fold rotational symmetry, it may not strike the eye as particularly anisotropic. Indeed, the magnitude of the Fermi velocity of PdCoO_2_ is almost constant around the Fermi surface^16^. The key aspect of directional ballistics, however, is the strong angle dependence of the velocity direction distribution, that is, the probability of finding a certain direction of quasiparticle velocity (Supplementary Note 4). As the nearly flat Fermi surface segments host large densities of states propagating essentially into the same direction, the velocity direction distribution is extremely anisotropic despite the relatively isotropic appearance of the Fermi surface. We propose such a velocity direction distribution map as a tool to visualize the propensity of a material to exhibit directional ballistics.

It is possible to go beyond the above qualitative discussion and perform Boltzmann transport simulations (see Supplementary Note 5 for details) which model the observations remarkably well. Using a Fermi surface shape parameterization established from angle-resolved photoemission data^28^, assuming diffusive boundary scattering and obtaining the temperature-dependent bulk mean free path from data on the 155-µm-wide sample (Supplementary Fig. S3) the simulations produce the results shown in Fig. 1d. We emphasize that this agreement is achieved without the use of any free parameters. To investigate the role of the various ingredients to this directional transport separately, we performed further calculations on more restricted models. First, a Boltzmann calculation including realistic bulk scattering but using an unrealistic, mathematically hexagonal Fermi surface probes the role of the rounded Fermi surface (Supplementary Note 6). A second Landau–Büttiker-type calculation includes a realistic Fermi surface model but ignores the bulk scattering (Supplementary Note 7). While both attempts reproduce some qualitative features of the data, the excellent match between simulation and experiment shown in Fig. 1d is only achieved by accounting simultaneously for the bulk scattering and the realistic Fermi surface.

### Transverse voltages in zero field

**Fig. 4:**
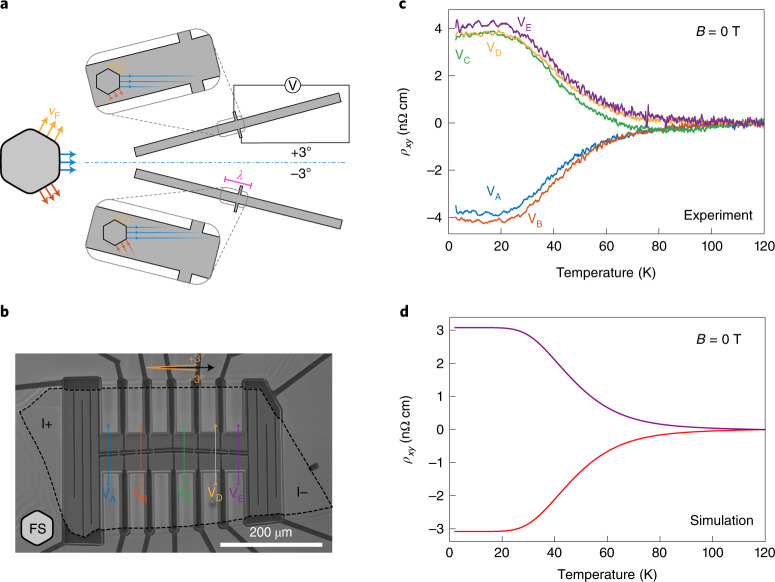
Temperature-dependent zero-field transverse voltage in PdCoO_2_. **a**, Sketch of several representative electronic trajectories in two transport bars cut along low-symmetry directions of the crystal, for example, ±3°. The corresponding orientation of the Fermi surface and velocities are shown on the left in yellow, blue and red. **b**, An FIB microstructured single crystal of PdCoO_2_ with a current path cut along the ±3° directions with three pairs of transverse voltage contacts along each orientation, denoted V_A_, V_B_, V_C_, V_D_ and V_E_. **c**, The extracted transverse resistivities increase from the noise floor in the diffusive regime to a finite and anti-symmetric value in the ballistic regime. A longitudinal background stemming from non-ideal transverse contact alignment (about 100 nm displacement) has been subtracted. The raw data, sample dimensions and corresponding analysis are presented in Supplementary Note 9. **d**, Results from Boltzmann transport simulations taking into account the realistic Fermi surface shape as well as the temperature-dependent bulk mean free path (see Supplementary Note 5 for details). Source data

Thus far, we have been concerned with directional ballistic effects observable in the longitudinal electrical transport. However, due to the broken rotational symmetry at the boundaries, finite off-diagonal terms are allowed in the conductivity matrix along low-symmetry directions. Such terms have been used to effectively probe bulk symmetry lowering in Ba(Fe_1−__*x*_Co_*x*_)_2_As_2_ single crystals^29^ and ﻿La_2−__*x*_Sr_*x*_CuO_4_ thin films^30^. In PdCoO_2_, these are expected because of directional microstructuring and can be accessed by the device geometry outlined in Fig. 4a. Along a low-symmetry direction, a transverse voltage in the ballistic regime develops due to an imbalance of electrons propagating towards the two different sides of the transport channel. Consequently, one expects the transverse voltage to be of equal strength but opposite sign with respect to the angle tilted away from a high-symmetry direction.

This can be tested in a specifically designed microstructure of PdCoO_2_ (Fig. 4b). The outline of the crystal is indicated by a dashed line. Following long current-homogenizing meanders at the injection (Supplementary Note 8), the heart of the sample consists of two serial transport bars cut at +3° and −3° with respect to the 0° direction, both 2.2 µm wide. Each of the bars has three pairs of opposite voltage contacts, allowing for simultaneous transverse and longitudinal voltage measurements. The resulting temperature dependence of the transverse zero-field resistivities *ρ*_*xy*_ is presented in Fig. 4c. In the diffusive transport regime at high temperatures, the transverse resistivity is absent, as expected after appropriate subtraction of the longitudinal voltage contribution originating from imperfect voltage contact alignment (see Supplementary Note 3 for details). With the dominant scattering off phonons in the bulk, the point-group symmetry of the material dominates the scattering, hence in-plane isotropy is symmetry enforced. However, upon entering the ballistic regime, a finite and asymmetric voltage develops across the device depending on the orientation of the transport bar. Again, the observations are closely reproduced by the Boltzmann simulation (Fig. 4d).

## Discussion

The excellent agreement between the experimental results presented here and the Boltzmann transport simulation of the directional ballistics highlights the importance of taking the Fermi surface shape and channel direction into account in the analysis of data from width-restricted channels of materials such as PdCoO_2_ with faceted Fermi surfaces. The analysis presented in ref. ^25^ considered a circular Fermi surface and hence treated the orientation of the channel relative to the crystal axes as unimportant. The results presented here show that, to conclusively identify a viscous contribution to transport in PdCoO_2_, further experiments on transport bars aligned along both the 0° and 30° directions will be required, combined with analysis using realistic models of hydrodynamic transport in which the faceting of the Fermi surface is taken into account^31–33^.

## Conclusions and outlook

We have shown, for the first time in a two-dimensional metal, that a strongly faceted Fermi surface can lead to strongly orientation-dependent conduction in otherwise identical ballistic devices cut from the same single crystal. These observations are of fundamental and practical importance to the question of the minimal attainable resistance in nanoscopic conductors, which ultimately limits the potential miniaturization of electric conductors in technological applications. As conductors are scaled down, even technologically relevant thin films enter the ballistic transport regime at elevated temperature, and boundaries become an important source of scattering^14^. Our results demonstrate that the boundary scattering contribution in zero field can be reduced by over a factor of two when a 2.5-µm-wide channel is aligned with one of the main directions of quasiparticle propagation. This is not a fundamental limit. Indeed, the smallest width we have studied in these proof-of-principle experiments is at least an order of magnitude larger than the minimum that could be envisaged. For wires less than 10 µm long, narrowed to widths of order 100 nm, the effect may be much larger (see the discussion in Supplementary Note 5), resulting in significant gains in attainable channel conductivity compared with that available from materials with circular Fermi surfaces. Our results invite investigation of other delafossite metals in which there are subtle differences in the degree of Fermi surface faceting^22^ and also a thorough study of the effects of magnetic field on directional ballistics.

Finally, we note that the phenomena we report here are far from being restricted to delafossites. Materials with facetted Fermi surfaces are not rare. Gated bilayer graphene^34^ is one of the most promising platforms for extensions of this research. While the Fermi surface anisotropy can also be controlled in GaAs-based quantum wells^35,36^, despite its anisotropic appearance, the Fermi velocity distribution remains quite isotropic. Most strikingly, the in-plane transport anisotropy is a widely used technique to detect subtle symmetry-lowering electronic states such as electronic nematicity. The implicit assumption is based on the group-theoretical argument that the appearance of an in-plane anisotropy in transport necessitates that the rotational symmetry of the bulk material be reduced to two-fold. Our work shows that, especially in clean metallic crystals, even symmetry lowering by the sample shape itself can induce such an anisotropic response, without any broken rotational symmetries in the point group of the unit cell. As mean free paths can indeed become macroscopic, these effects may well appear in traditional single crystals. This may prove to be important in the interpretation of unconventional transport phenomena in topological semi-metals, which generally tend to be of high mobility. For example, a mean free path *λ* > 100 µm is readily observed in the Weyl II semi-metal WP_2_ (ref. ^37^), which in turn implies that even the sub-mm-sized single crystals used in traditional conductivity measurements are in a quasi-ballistic transport regime. Given the common deviation from circular Fermi surfaces in this materials class, the effects uncovered here are likely to be of relevance to that field.

It will also be interesting to consider whether directional ballistics plays a role in generating subtle symmetry-forbidden transport signals at phase transitions of strongly correlated materials, in which one might a priori not expect to encounter ballistic behaviour due to their short mean free path. However, because the most strongly interacting states at the Fermi level are those driving the ordered phase, the average lifetime of the remaining quasiparticle states often increases significantly upon electronic ordering. Famous examples of dramatic enhancements of the mean free path include the hidden order transition in URu_2_Si_2_^38^, the nodal quasiparticles in YBa_2_Cu_3_O_7_^39^ and the formation of coherent heavy fermion liquids^40^. It is interesting to note that all these microscopically distinct correlated transitions are associated with weak symmetry-forbidden anisotropies, and at the same time these phase transitions push these materials closer to the directional ballistics limit. In summary, we believe that much remains to be investigated concerning the physics as well as potential applicability of directional ballistics.

## Online content

Any methods, additional references, Nature Research reporting summaries, source data, extended data, supplementary information, acknowledgements, peer review information; details of author contributions and competing interests; and statements of data and code availability are available at 10.1038/s41567-022-01570-7.

## Supplementary information

Supplementary information Supplementary Notes 1–9 and Figs. 1–9.

Supplementary data Source data for Supplementary Figs. 1, 3, 4, 5, 6, 8 and 9c–f.

## Data Availability

All raw data underpinning this publication can be accessed in comprehensible ASCII format at 10.5281/zenodo.5964955. Source data are provided with this paper.

## Source data

Source Data Fig. 1 Source data of Fig. 1b–d.
Source Data Fig. 4 Source data of Fig. 4b,d.

## Code availability

Source code for the simulations can be found at https://github.com/dgglab/ballistic_montecarlo.
