# Supplementary material for: Directional ballistic transport in the two-dimensional metal PdCoO2
Source: Nat Phys. 2022 May 9;18(7):819–24. doi: 10.1038/s41567-022-01570-7 (PMC9279146; doi:10.1038/s41567-022-01570-7)
Supplement: Supplementary file 1 — Supplementary Notes 1–9 and Figs. 1–9. [file 41567_2022_1570_MOESM1_ESM.pdf]

nature portfolio

https://doi.org/10.1038/s41567-022-01570-7

Supplementary information

Directional ballistic transport in the two-dimensional metal $PdCoO_2$

In the format provided by the
authors and unedited

Supplementary Information for Bachmann *et al.* "Directional ballistic transport in the two-dimensional metal $PdCoO_2$"

Supplementary Note 1| 2D conductivity tensors on square, triangular and hexagonal crystal lattices

On the Cartesian basis, where $\hat{x}$ and $\hat{y}$ define the plane of the 2D lattice, the generic conductivity tensor C can be written as

$$C = \begin{pmatrix} \sigma_{xx} & \sigma_{xy} \\ \sigma_{yx} & \sigma_{yy} \end{pmatrix}.$$

When the symmetries of the respective crystal system are imposed onto C, the number of independent components is reduced. We are concerned with the dihedral point groups D_n with $n = 4{,}6$, which have an n-fold rotation axis and n two-fold reflection axes perpendicular to the rotation axis. The symmetry operations describing the rotation and reflection can be represented in matrix form. The rotation matrix $R(\theta)$, which expresses a counterclockwise rotation through an angle θ around the out-of-plane axis is given by

$$R(\theta) = \begin{pmatrix} \cos\theta & -\sin\theta \\ \sin\theta & \cos\theta \end{pmatrix}$$

The rotation matrix is orthogonal, i.e. it has the property $R^{\mathrm{T}}R = RR^{\mathrm{T}} = \mathbb{I}$. The rotated conductivity matrix C' is obtained by calculating $C' = R(\theta) \cdot C \cdot R^{\mathrm{T}}(\theta)$.

The matrix describing reflection about the *y*-axis, for instance, is given by

$$S = \begin{pmatrix} -1 & 0 \\ 0 & 1 \end{pmatrix}$$

and the reflected conductivity matrix can be found by calculating $C' = S \cdot C \cdot S^{\mathrm{T}}$. Imposing this reflection symmetry onto the general conductivity tensor C by requiring $C' = C$, we find $\sigma_{xy} = \sigma_{yx} = 0$ and can hence exclude off-diagonal elements.

Further, considering the case of the square lattice, which is invariant under rotation about $\theta = \pi/2$, imposing C_4 symmetry on to the conductivity matrix ($C' = R(\pi/2) \cdot C \cdot R^{\mathrm{T}}(\pi/2)$) yields $\sigma_{xx} = \sigma_{yy} \equiv c$ and $\sigma_{xy} = -\sigma_{yx}$. With the reflection symmetry requiring $\sigma_{xy} = \sigma_{yx} = 0$ we find that the general conductivity matrix on a square lattice is given by $C = c \cdot \mathbb{I}$, where c is a scalar and $\mathbb{I}$ is the unitary matrix.

The same formalism can be repeated in the case of a triangular or hexagonal lattice, which are invariant under rotations about $\theta = 2\pi/3$ or $\theta = \pi/3$ respectively. Following the above procedure, one again finds that the conductivity matrices can be expressed by $C = c \cdot \mathbb{I}$.

It is then straightforward to see that in any system, in which the conductivity matrix can be expressed as a scalar multiplied with the identity matrix must have an isotropic conductivity[41].

Supplementary Note 2| Damage due to ion beam irradiation

The FIB does induce surface damage in the outermost approximately 20nm, but in $PdCoO_2$ does not lead to any further propagation of defects into the bulk. Previous studies[15,25] have demonstrated that beyond the expected thin damage layer of 20nm adjacent to a FIB-cut surface, there is no resolvable effect either on residual resistivity or on the amplitude of quantum oscillations, which are bulk probes of sample purity.

Supplementary Note 3| Comparison of the temperature dependent resistivity for devices of different widths.

The temperature dependent resistivity of $PdCoO_2$ can directly be converted into an estimate for the electron mean free path λ using the standard 2D expression[42]:

$$\rho^{-1} = \frac{e^2}{hd} k_{\mathrm{F}} \lambda$$

where ρ is the in-plane resistivity, e is the electron charge, h is the Planck constant, d=17.73/3Å is the palladium layer separation, k_{F} = 0.95/Å is the average Fermi momentum. The average is calculated by integrating over the entire Fermi surface: $k_{\mathrm{F}} = \frac{1}{2\pi}\int_0^{2\pi} |k_F(\theta)|\, d\theta = 0.952\,\text{Å}^{-1}$, where a parametrization of the Fermi surface can be found here[28].

Here we display and compare the resistivities and resultant mean free path estimations for 3 $PdCoO_2$ devices with widths w = 155μm, 7μm, and 2.5μm. The data for the 155μm wide sample have been replotted from reference[16] and are available at https://edmond.mpdl.mpg.de/imeji/collection/MWrmtAADcp5wH6Yx.

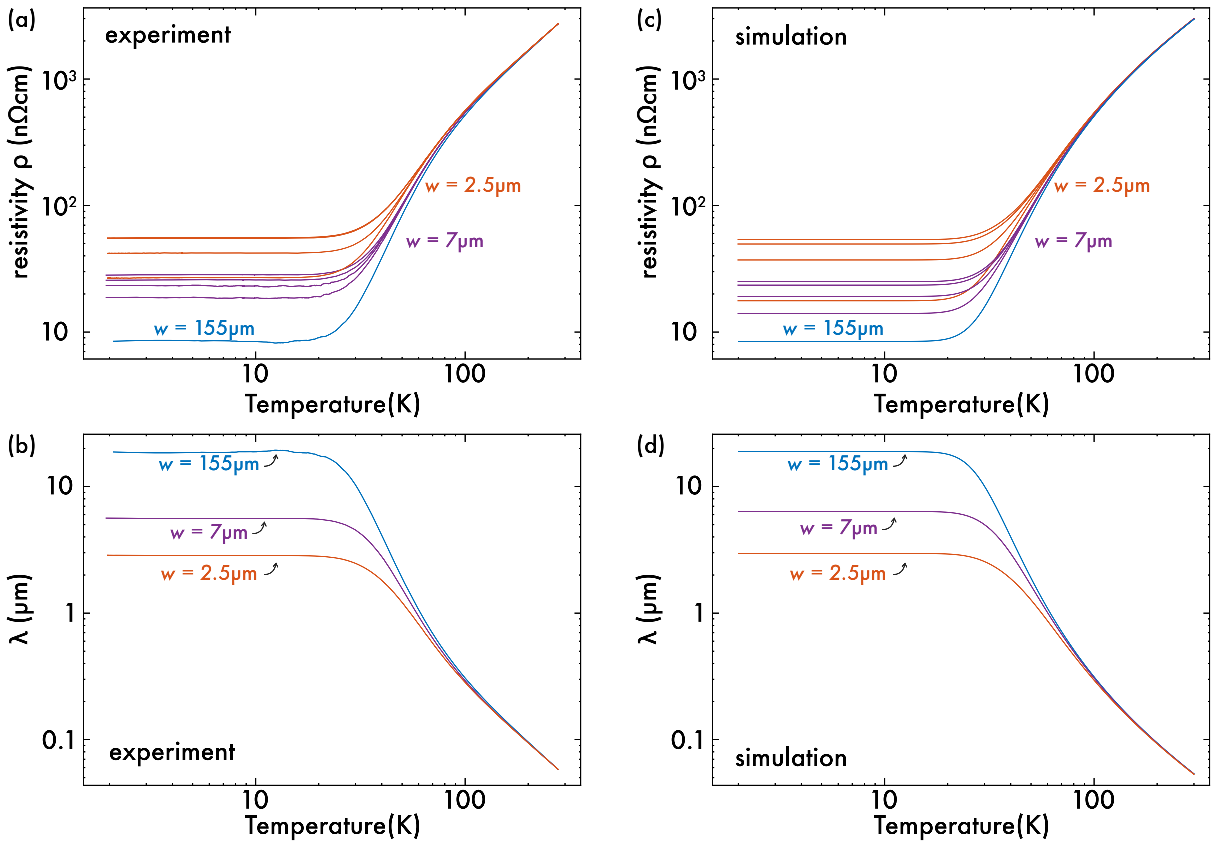

Fig. S1| Comparison of the temperature dependence of the resistivity and mean free path in $PdCoO_2$ devices of different widths. (a) The resistivity as a function of temperature for 3 different devices with widths of 2.5μm, 7μm and 155μm (data for 155μm wide sample taken from reference[16]). For the narrow devices, the resistivities along the 0°, 10°, 20°, and 30° direction are displayed. While the 155 μm device is assumed to be in the bulk limit over the entire temperature range, the narrow devices enter the ballistic regime at low temperatures. The temperatures at which the mean free path λ is equal to the device width w is estimated to be 46 K and 34 K for the 2.5 μm and 7 μm wide samples respectively. These temperatures are consistent with an in-plane anisotropy developing in the devices as evidenced by the curves fanning apart at low temperatures. (b) The temperature dependent mean free path λ calculated directly from the in-plane resistivity along the 30° direction. While the electrons in the bulk sample (w = 155μm) are not influenced by boundary scattering events, the curves associated with the 2.5μm and 7μm devices are truncated by the device widths rather than the intrinsic bulk mean free path. (c) and (d) Results from Boltzmann transport simulations taking into account the realistic Fermi surface shape as well as the temperature dependent bulk mean free path. See Supplementary Note 5 for details.

Supplementary Note 4| Calculation of the Fermi velocity direction distribution

Here we describe the derivation of the angular distribution of the Fermi velocity direction based on an angularly parametrized Fermi surface.

From precise ARPES measurements[28], the in-plane Fermi surface of $PdCoO_2$ is well known and can be expressed as a periodic function of angle φ:

$$\begin{pmatrix} k_F^x \\ k_F^y \end{pmatrix} = k_0 \begin{pmatrix} \cos\varphi \\ \sin\varphi \end{pmatrix} + k_6 \cos 6\varphi \begin{pmatrix} \cos\varphi \\ \sin\varphi \end{pmatrix} + k_{12} \cos 12\varphi \begin{pmatrix} \cos\varphi \\ \sin\varphi \end{pmatrix}$$

where the values k_0 = 0.9518Å^{-1}, k_6 = 0.0444Å^{-1}, k_{12} = 0.0048Å^{-1} are taken from[28]. The corresponding curve is displayed in figure S6.

Next, the Fermi surface must be expressed by points separated by constant arc length. The arc length, s, as a function of polar angle is given by $s(\varphi) = \sqrt{(\partial_x k_F^x(\varphi))^2 + (\partial_y k_F^y(\varphi))^2}$. With this, the Fermi surface curve can be interpolated, such that it is spanned by points of equal arc length. This is straightforward to implement numerically. Next, we set out to find the distribution of the Fermi velocity direction, θ, in Fig. S6, as a function of polar angle φ.

In general, the direction of a normal vector at any point along a parametric curve $\mathbf{r}(\varphi) = (\mathbf{x}(\varphi), \mathbf{y}(\varphi))$ can be found by first taking the derivative $\partial\mathbf{r}/\partial\varphi = (\partial\mathbf{x}/\partial\varphi, \partial\mathbf{y}/\partial\varphi)$, which yields the tangent vector to the curve and then rotating it clockwise by 90 degrees. This results in a vector $\widetilde{v_F} = (\partial\mathbf{y}/\partial\varphi, -\partial\mathbf{x}/\partial\varphi)$ which is parallel to the Fermi velocity. Finally, the velocity direction is simply given by the angle θ of the rotated tangent vector, which can be found by calculating $\theta(\varphi) = \tan^{-1}\left(-\frac{\partial \mathbf{x}}{\partial\varphi} / \frac{\partial \mathbf{y}}{\partial\varphi}\right)$. This calculation is displayed graphically in Fig. S6.

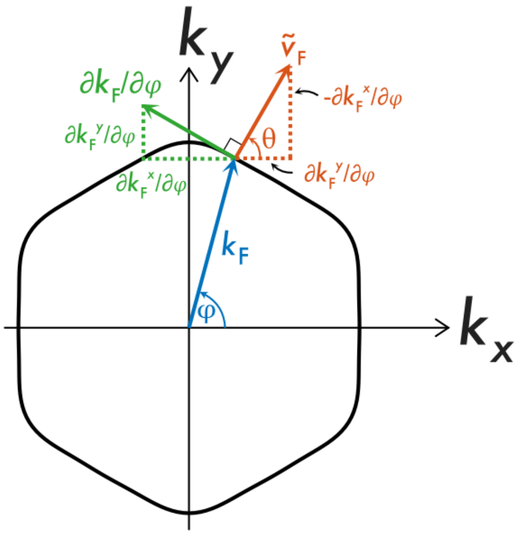

Fig. S2|Derivation of the Fermi velocity direction distribution. In order to determine the Fermi velocity distribution the direction of the Fermi velocity, θ, as a function of polar angle φ must be calculated.

Finally, the angular Fermi velocity direction distribution is found by evaluating the function $\theta(\varphi)$ at equidistant arc lengths along the Fermi surface and displaying the normalized probability density as a function of polar angle, as is presented in Fig. 3.

Supplementary Note 5 | Boltzmann transport simulations including realistic Fermi surface shape and bulk scattering

We develop a model for electrical conduction in a finite-width channel for an anisotropic metal with arbitrary Fermi surface. We proceed by analogy with the results of reference[27], which treated the case of an isotropic, free-electron metal. We apply this model to the present data by using a Fermi surface parametrization for $PdCoO_2$ coming from ARPES measurements[28] and by extracting a temperature-dependent bulk mean free path from bulk resistivity measurements[16].

We consider a channel of width W extending from $y = 0$ to $y = W$. We write the distribution function as

$$f_{\boldsymbol{k}}(y) = f_0 + \delta f_{\boldsymbol{k}}(y)$$

where f_0 is the equilibrium distribution function and $\delta f_{\boldsymbol{k}}(y)$ represents a small deviation from equilibrium. The linearized Boltzmann equation in the relaxation time approximation and in the presence of an electric field $\boldsymbol{E}$ is given by

$$v_{\boldsymbol{k}y} \frac{\partial\, \delta f_{\boldsymbol{k}}(y)}{\partial y} + \frac{e}{\hbar} \boldsymbol{E} \cdot \boldsymbol{v}_{\boldsymbol{k}} \left(-\frac{\partial f_0}{\partial \mathcal{E}_{\boldsymbol{k}}}\right) = -\frac{1}{\tau} \delta f_{\boldsymbol{k}}(y)$$

where

$$\boldsymbol{v}_{\boldsymbol{k}} = \frac{1}{\hbar} \nabla_{\boldsymbol{k}} \mathcal{E}_{\boldsymbol{k}}.$$

We assume diffuse scattering at the boundaries of the channel. Using $\delta f_{\boldsymbol{k}}^{+(-)}$ to denote the non-equilibrium distribution function of electrons with $v_{\boldsymbol{k}y} > 0$ ($v_{\boldsymbol{k}y} < 0$), this imposes the conditions

$$\begin{aligned} \delta f_{\boldsymbol{k}}^{+}(0) &= 0 \\ \delta f_{\boldsymbol{k}}^{-}(W) &= 0. \end{aligned}$$

This has the solution

$$\delta f_{\boldsymbol{k}}(y) = \frac{e\tau}{\hbar} \boldsymbol{E} \cdot \boldsymbol{v}_{\boldsymbol{k}} \left(-\frac{\partial f_0}{\partial \mathcal{E}_{\boldsymbol{k}}}\right) g_{\boldsymbol{k}}(y)$$

where

$$g_{\boldsymbol{k}}(y) = \begin{cases} 1 - \exp\left(-\dfrac{y}{\tau v_{\boldsymbol{k}y}}\right) & v_{\boldsymbol{k}y} > 0, \\ 1 - \exp\left(\dfrac{W - y}{\tau v_{\boldsymbol{k}y}}\right) & v_{\boldsymbol{k}y} < 0. \end{cases}$$

Electrical current is given by

$$\boldsymbol{J} = -2e \int \frac{d\boldsymbol{k}}{(2\pi)^3} \boldsymbol{v}_{\boldsymbol{k}}\, \delta f_{\boldsymbol{k}}.$$

We define a spatially-dependent conductivity tensor by

$$\sigma_{ij}(y) \equiv \frac{J_i(y)}{E_j}.$$

Let $S(\mathcal{E})$ be the surface of constant energy defined by $\mathcal{E} = \mathcal{E}_{\boldsymbol{k}}$. Then $d\boldsymbol{k} = d\mathcal{E}dS/\hbar v_{\boldsymbol{k}}$. Since $T \ll T_F$, we have that $(-\partial f_0/\partial\mathcal{E}_{\boldsymbol{k}}) \to \delta(\mathcal{E}_{\boldsymbol{k}} - \mathcal{E}_F)$. Then we get

$$\sigma_{ij}(y) = \frac{e^2\tau}{4\pi^3}\int_{S_F} dS \frac{v_{\boldsymbol{k}i}v_{\boldsymbol{k}j}}{v_{\boldsymbol{k}}}\, g_{\boldsymbol{k}}(y)$$

where $S_F = S(\mathcal{E}_F)$ and $v_{\boldsymbol{k}} = |\boldsymbol{v}_{\boldsymbol{k}}|$. Next we define a spatial average by

$$\bar{A} = \frac{1}{W}\int_0^W dy\, A(y)$$

and obtain a spatially-averaged conductivity tensor as

$$\bar{\sigma}_{ij} = \frac{e^2\tau}{4\pi^3}\int_{S_F} dS\, \frac{v_{\boldsymbol{k}i}v_{\boldsymbol{k}j}}{v_{\boldsymbol{k}}}\, \bar{g}_{\boldsymbol{k}}$$

where

$$\bar{g}_{\boldsymbol{k}} = 1 - \frac{\tau|v_{\boldsymbol{k}y}|}{W}\left[1 - \exp\left(-\frac{W}{\tau|v_{\boldsymbol{k}y}|}\right)\right].$$

Resistivity is found by inverting the spatially-averaged conductivity tensor:

$$\rho_{ij} = \bar{\sigma}_{ij}^{-1}.$$

The bulk resistivity tensor ρ_{ij}^{bulk} is found from the preceding equations by setting $\bar{g}_{\boldsymbol{k}} = 1$. As per the discussion in Supplementary Note 1, the bulk resistivity tensor for $PdCoO_2$ within a two-dimensional approximation has a single unique and non-zero component, ρ_{xx}^{bulk}.

For a Fermi surface S_F parametrized by the Fermi vector $\boldsymbol{k}_F(g,h)$, we define a vector

$$\boldsymbol{n}(g,h) = \frac{\partial\boldsymbol{k}_F(g,h)}{\partial g} \times \frac{\partial\boldsymbol{k}_F(g,h)}{\partial h}.$$

Then the integral over the Fermi surface is given by

$$\int_{S_F} dS = \int_{g_1}^{g_2} dS \int_{h_1}^{h_2} dh\, n(g,h)$$

where $n(g,h) = |\boldsymbol{n}(g,h)|$. The unit vector normal to the Fermi surface—and thus parallel to the Fermi velocity—is given by

$$\hat{\boldsymbol{n}}(g,h) = \frac{\boldsymbol{n}(g,h)}{n(g,h)}.$$

For $PdCoO_2$, in a two-dimensional approximation, the Fermi surface can be parametrized as

$$\boldsymbol{k}_F(\phi,\phi_0,k_z) = k_F(\phi - \phi_0 - \pi/2)[\cos\phi\,\hat{\mathbf{i}} + \sin\phi\,\hat{\mathbf{j}}] + k_z\hat{\mathbf{k}}$$

where ϕ_0 is the channel orientation as labelled in Fig. 1a, and with

$$k_F(\phi) = \sum_\mu k_\mu \cos(\mu\phi)$$

where the k_μ are listed in Supplementary Note 4. Furthermore, $\phi \in (0,2\pi)$ and $k_z \in (-\pi/d, \pi/d)$ with d =17.743/3 Å. We assume that the Fermi velocity has a constant magnitude v_F:

$$\boldsymbol{v_k} = v_F \hat{\boldsymbol{n}}_{\boldsymbol{k}}$$

for $\boldsymbol{k}$ on S_F, as justified by electronic structure calculations[16] and ARPES measurements[28]. We use[17] $v_F = 7.5\times10^5$ m/s.

Using only the shape of the Fermi surface, we can calculate $\rho_{ij}/\rho_{xx}^{\text{bulk}}$, the ratio of the resistivity ρ_{ij} of a finite channel to the longitudinal resistivity ρ_{xx}^{bulk} of an infinite channel. This depends only on W/λ, the ratio of the channel width W to the bulk mean free path $\lambda = v_F\tau$. Fig. S5 displays this ratio as a function of channel direction for two values of W/λ.

To make a direct comparison with experimental data, it is necessary to determine the temperature-dependent bulk relaxation time $\tau(T)$. To do so, we fit the temperature-dependent resistivity of the 155 µm channel, which we assume to be in the bulk limit. We fit to a phenomenological temperature dependence

$$\rho_{xx}^{\text{bulk}}(T) = \rho_\alpha + \frac{\rho_\beta}{e^{T_0/T} - 1}.$$

The second term, which has a Bose–Einstein-like form, was motivated by the observed $\rho_{xx}^{\text{bulk}} \sim e^{-T/T_0}$ behavior at low temperature[17] and $\rho_{xx}^{\text{bulk}} \sim T$ behavior at high temperature. The fit is shown in Fig. S4. We then determined $\tau(T)$ from the fit to $\rho_{xx}^{\text{bulk}}(T)$ using the bulk limit of the above equations for the conductivity.

To extract the experimental transverse resistivity data presented in Fig. 4c, a longitudinal background had to be subtracted from the raw data, as explained in Supplementary Note 3. In doing so, it was assumed that the transverse resistivity is zero at 120 K. To treat the data and calculation equally, the calculation presented in Fig. 4d was obtained by subtracting a constant value such that the transverse resistivity was set to zero at 120 K.

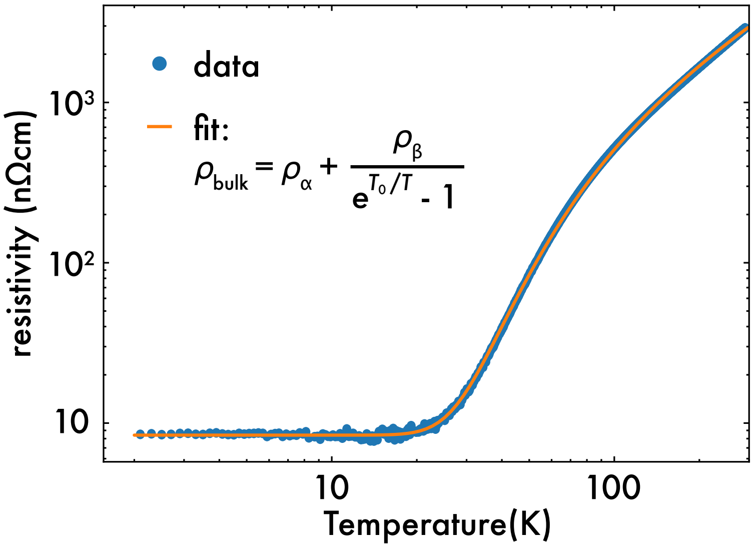

Fig. S3|Fit of the bulk resistivity as a function of temperature curve. The bulk resistivity data for a 155μm wide transport device was obtained and replotted from reference[16]. Using the fit function in the figure, we obtain the fit parameters ρ_α = 8.41nΩcm, ρ_b = 2.29 μΩcm, T_0 = 172K.

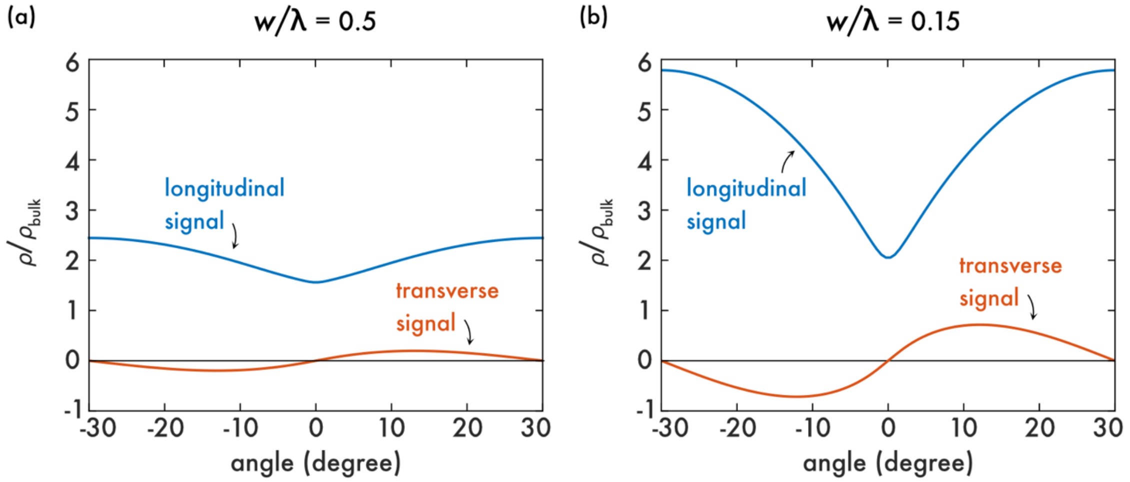

Fig. S4| Calculated longitudinal and transverse resistivity anisotropy for a realistic $PdCoO_2$ Fermi surface. For two ratios of w/λ, specifically (a) w/λ = 0.5 and (b) w/λ = 0.15.

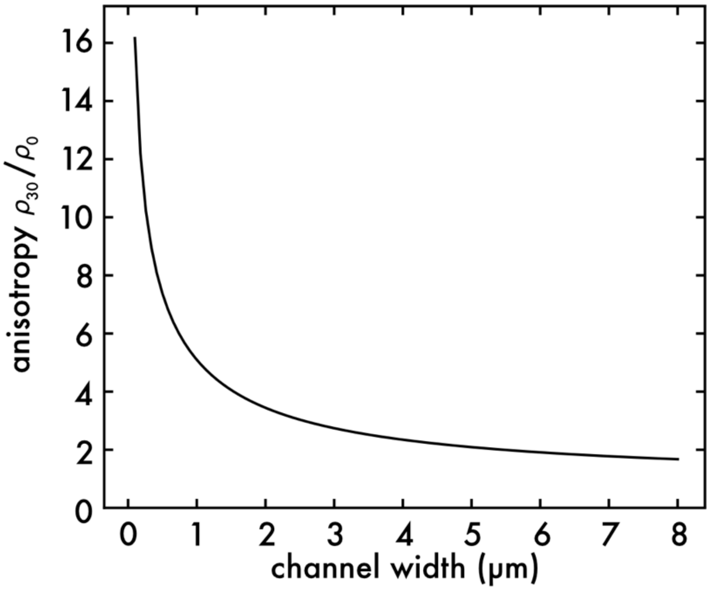

Fig. S5| Predicted resistivity anisotropy from realistic Boltzmann transport simulations. The simulations were performed for transport bars along the 0- and 30-degree direction. The ratio of the resistance increases between the 30- and 0-degree direction is shown above and is proportional to the resistive anisotropy. In line with intuition, this anisotropy is predicted to grow rapidly with decreasing channel width. These predictions are for a sample of infinite length; if the length also becomes of order the mean free path, the intuitive expectation is for a even larger anisotropies than those shown here.

Supplementary Note 6| Kinetic calculations for a perfectly hexagonal Fermi surface

We solved the linearized Boltzmann equation in the relaxation time approximation with a perfectly hexagonal Fermi surface. We considered the case of an infinitely long wire along the x direction, and of finite section W along the y direction. The relative direction of the crystalline axis with respect to x is defined by the angle θ, such that $\theta = -\pi/6$ corresponds to the "angle" defined in the main text being 0. Diffuse scattering is assumed at the two boundaries of the wire, i.e. at $y = \pm w/2$.

We denote the out-of-equilibrium distribution of quasi-particles by $\chi(\boldsymbol{k}, y)$, and the mean free path by λ. It is easy to see that, for the geometry considered, $\chi(\boldsymbol{k}, y)$ takes a uniform value on each of the 6 edges of the Fermi surface. We therefore use the notation $\chi_j(y)$, where j is an index of the Fermi surface edge such that the edge j has velocity $\cos(\phi_j)\,\hat{x} + \sin(\phi_j)\,\hat{y}$ with $\phi_j = \left[\frac{\pi}{6}, \frac{3\pi}{6}, \frac{5\pi}{6}, \frac{7\pi}{6}, \frac{9\pi}{6}, \frac{11\pi}{6}\right] - \theta$.

The linearized Boltzmann equation takes the form

$$\sin\phi_j \partial_j \chi_j + \frac{\chi_j}{l} = \frac{\bar{\chi}}{l}$$

with $\bar{\chi} = \frac{1}{6}\sum_j \chi_j$.

The boundary conditions are

$$\chi_{1,2,3}(y = -w/2) = +l\cos\phi_j + \chi_b$$
$$\chi_{1,2,3}(y = +w/2) = +l\cos\phi_j + \chi_t$$

where χ_b and χ_t should be chosen so as to satisfy $j_y = 0$.

The density and the currents are given by

$$n = \bar{\chi}$$

$$j_x = \frac{1}{6}\sum_j \chi_j \cos(\phi_j)$$

$$j_y = \frac{1}{6}\sum_j \chi_j \sin(\phi_j)$$

The solution of the Boltzmann equation is

$$\chi_{j=1,2,3} = \chi_j\left(y = -\frac{W}{2}\right)e^{-\frac{y+\frac{W}{2}}{l\,\sin(\phi_j)}} + \int_{-\frac{W}{2}}^{\infty} dy'\Theta(y-y')e^{-\frac{(y-y')}{l\,\sin(\phi_j)}}\frac{\bar{\chi}(y')}{l\,\sin(\phi_j)}$$

$$\chi_{j=4,5,6} = \chi_j\left(y = \frac{W}{2}\right)e^{-\frac{y-\frac{W}{2}}{l\,\sin(\phi_j)}} - \int_{-\infty}^{W/2} dy'\Theta(y'-y)e^{-\frac{(y-y')}{l\,\sin(\phi_j)}}\frac{\bar{\chi}(y')}{l\,\sin(\phi_j)}$$

Proof:

$$\partial_y \chi_{j=1,2,3} = \int_{-\frac{w}{2}}^{\infty} dy' \left(\delta(y-y') e^{-\frac{(y-y')}{l\,\sin(\phi_j)}} \frac{\bar{\chi}(y')}{l\,\sin(\phi_j)} + \Theta(y-y') e^{-\frac{(y-y')}{l\,\sin(\phi_j)}} \frac{\bar{\chi}(y')}{l\sin(\phi_j)} \frac{-1}{l\sin(\phi_j)} \right)$$
$$= \frac{\bar{\chi}(y)}{l\sin(\phi_j)} - \frac{1}{l\sin(\phi_j)} \chi_j$$

For numerical reasons, it is convenient to use

$$F(y_2) = \int_{-\frac{w}{2}}^{y_2} dy'\, e^{-\frac{(y_2-y')}{l\,\sin(\phi_j)}} \frac{\bar{\chi}(y')}{l\sin(\phi_j)} = e^{(y_1-y_2)/l/\sin(\phi_j)} F(y_1) + \int_{y_1}^{y_2} dy'\, e^{-\frac{(y_2-y')}{l\,\sin(\phi_j)}} \frac{\bar{\chi}(y')}{l\sin(\phi_j)}$$

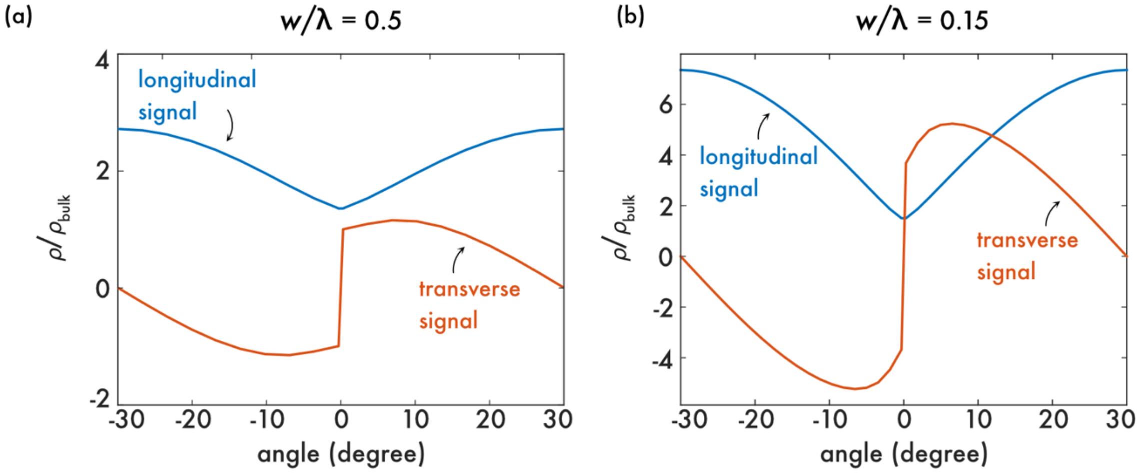

Fig. S6|Calculated longitudinal and transverse resistivity anisotropy for a perfectly hexagonal Fermi surface. For two ratios of w/λ, specifically (a) w/λ = 0.5 and (b) w/λ = 0.15. The anisotropies in the longitudinal anisotropy are 1.9 and 5, both overestimates compared to the experimentally measured values, with the discrepancy quite large for the narrower channel. This is likely to be a result of using the idealized perfect hexagon for the Fermi surface instead of a more realistic one with rounded corners. Unsurprisingly this also results in an overestimate of the ratio of transverse to longitudinal resistivity, predicted to lie in the range 0.8-2.6 for channels of the dimension shown in Fig. 3, compared with our measured value of 0.3. Nevertheless, the qualitative trends of our observations are well captured by the calculations.

Supplementary Note 7| Monte Carlo simulations based on Landauer-Büttiker formalism and realistic Fermi surface shape

We model the electron trajectories according to their semiclassical equations of motion for an out-of-plane magnetic field $\boldsymbol{B} = B\hat{\boldsymbol{z}}$:

$$\hbar v = \frac{\partial \varepsilon}{\partial k}, \qquad \hbar \dot{\boldsymbol{k}} = -e\boldsymbol{E} + eB\hat{z} \times \boldsymbol{v}$$

where $\hbar$ is the reduced Planck's constant, e is the charge of an electron, $\boldsymbol{v}$ is the Fermi velocity, and $\boldsymbol{E}$ is the electric field experienced by the electron. In the ballistic regime, there is negligible electric field in the bulk, therefore we assume that $\boldsymbol{E} = 0$. We take a tight binding approximation of the Fermi surface[17] based on ARPES data[18],

$$\boldsymbol{k_F}(\theta) = k_0 + k_6 \cos(6\theta) + k_{12} \cos(12\theta)$$

where $k_0 = 0.95\text{Å}^{-1}$, $k_6 = 0.05\ \text{Å}^{-1}$, and $k_{12} = 0.006\ \text{Å}^{-1}$. Because we are not concerned with transit times of the electrons, we can ignore the Fermi velocity v.

To model the transport anisotropy, the carriers are injected into a two-dimensional bar of width w and length L. Ohmic contacts are created at two ends of the bar which will serve as an injector and a ground. We will assume that the ground is perfect in that any electron colliding with this contact is absorbed and removed from the device. These electrons then follow their semi-classical path[43], ignoring bulk scattering, until interacting with either an edge or ohmic contact of the device. When injecting an electron into the system or scattering from an edge of the device, the probability of injecting into a state n of the discretized Fermi surface is

$$p(n) = cos(\theta(n) - \phi)$$

where $\theta(n) = tan(v_y/v_x)$ is the direction of propagation of the state n and ϕ is the angle of the normal to the edge. The Fermi surface is numerically discretized into states separated by constant arclength to remove the probability distribution's dependence on Fermi velocity[43]. The nearly perfectly hexagonal Fermi surface of $PdCoO_2$ has approximately flat edges which cause a high density of states to be injected at fixed angles. In the case of a non-ohmic edge, a carrier is scattered into a new state chosen according to the probability distribution for that edge. To ensure detailed-balance, if the injecting contact absorbs an incident carrier, the carrier is reemitted at a random position along the lead in a randomly chosen allowed state for that edge.

Electrons are injected uniformly across the edge of the contact into states chosen according to the probability distribution for the contact. Therefore, to estimate the two-terminal resistance of the bar, we calculate the forward-propagating flux through a line perpendicular to the channel several times the bars width from the injecting contact. The forward propagating flux is defined as

$$\phi_{forward} = \frac{1}{w}\left(N_{inject} + \frac{N_{cross} - N_{inject}}{2}\right)$$

where N_{cross} is the number of times charge carriers cross the line and N_{inject} is the number of injected carriers.

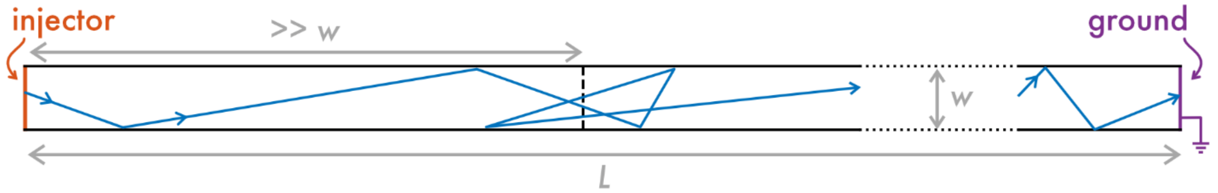

Fig. S7| Sketch of the simulation setup. Electrons propagate along a bar of length L and width w. The injector (left) and ground (right) are assumed to be perfect ohmic contacts. A virtual line placed a distance several times the width of the bar away from the injection point is used to calculate to forward propagating flux.

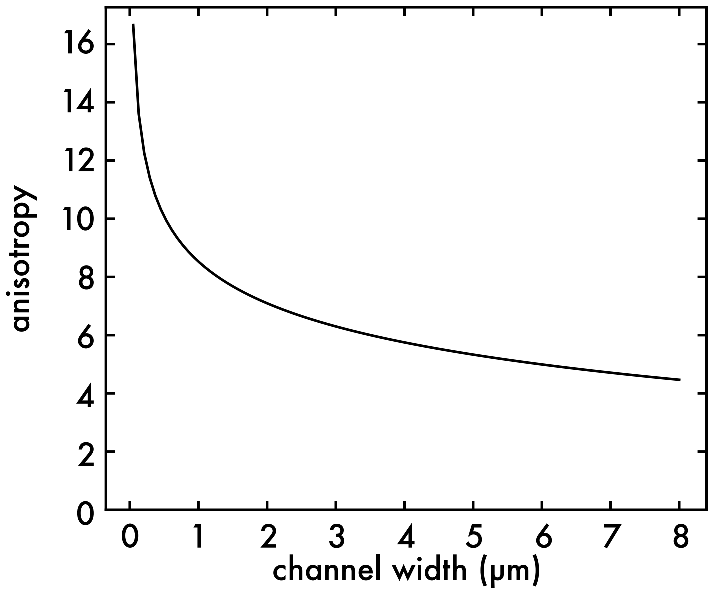

Fig. S8| Predicted resistivity anisotropy from Monte Carlo Simulations. Monte Carlo simulations were performed for transport bars along the 0- and 30-degree direction. The ratio of the resistance increases between the 30- and 0-degree direction is shown above and is proportional to the resistive anisotropy. In line with intuition, this anisotropy is predicted to grow rapidly with decreasing channel width.

Supplementary Note 8| Current homogeneity

Due to the large resistive anisotropy $\rho_{\text{out-of-plane}}$ / $\rho_{\text{in-plane}}$ which ranges up to a value of 2000 at low temperatures, care must be taken to ensure a homogeneous current flow through the device. Since the current into the device is injected through evaporated top-contacts, there must be enough length for the current to diffuse 'downwards' and flow parallel to the conducting layers. Through COMSOL simulations we have found that the minimum length required for the current to flow homogenously scales as $\sqrt{\rho_{out-of-plane}/\rho_{in-plane}} \cdot t$, where t is the device thickness. For the devices presented here in Fig. 1 and Fig. 4, the thickness of the devices is 7.8μm and 1.54μm respectively. In this case the minimum length required for the current injection meanders is 350μm and 70μm, which is satisfied in both cases.

Supplementary Note 9| Raw data and device dimensions of the measurements shown in Error! Reference source not found.4.

Typically, transverse voltage measurements are performed in a magnetic field, where the expected odd voltage response is found by anti-symmetrizing the measured voltage and thereby eliminating any undesired longitudinal (symmetric) component to the signal arising from contact misalignment. Here the measurement is performed in zero field and so this scheme cannot be used. Instead, we first ensured that opposing contacts are aligned as well as possible by in-situ determining the transverse voltage in the ohmic regime at room temperature in the FIB machine and minimizing the signal by using the ion beam to polish the voltage contacts accordingly. From the room temperature values in panels (c) and (d) as well as the device dimensions given listed in panel (b) we can deduce the that contact misalignment is at most 170nm. ($\Delta l = \frac{R}{\rho} \cdot w \cdot h = \frac{1.3\text{m}\Omega}{2.6\mu\Omega\text{cm}} \cdot 2.2\mu m \cdot 1.54\mu m = 170\text{nm}$). Further, in addition to the transverse voltages shown in panel (c), we have also simultaneously determined the longitudinal voltages presented in panel (d). This subsequently allowed us to scale the longitudinal voltages onto the transverse voltages, as indicated by the black curves in panel (e). This scaling is straightforward: For each curve we used a constant multiplication factor (specified in the figure caption) to scale the longitudinal resistances onto the transverse resistances such that the curves overlap at high temperatures, i.e. to find the appropriate multiplication factor m for a specific transverse resistance curve we calculated the ratio of the as-measured longitudinal to transverse resistances at high temperature:

$$m = R_{\text{longitudinal}}(300\text{K}) / R_{transverse}(300\text{K})$$

Then we multiply $R_{\text{longitudinal}}(T)$ by the constant m over the entire temperature range and subtract this from the as-measured $R_{transverse}$ (T):

$$R_{xy}(T) = R_{transverse}(T) - m^* R_{\text{longitudinal}}(T).$$

Finally, by multiplying the transverse resistance $R_{xy}(T)$ by the height, h, of the sample we find the sought for 'pure' transverse resistivity presented in panel (f).

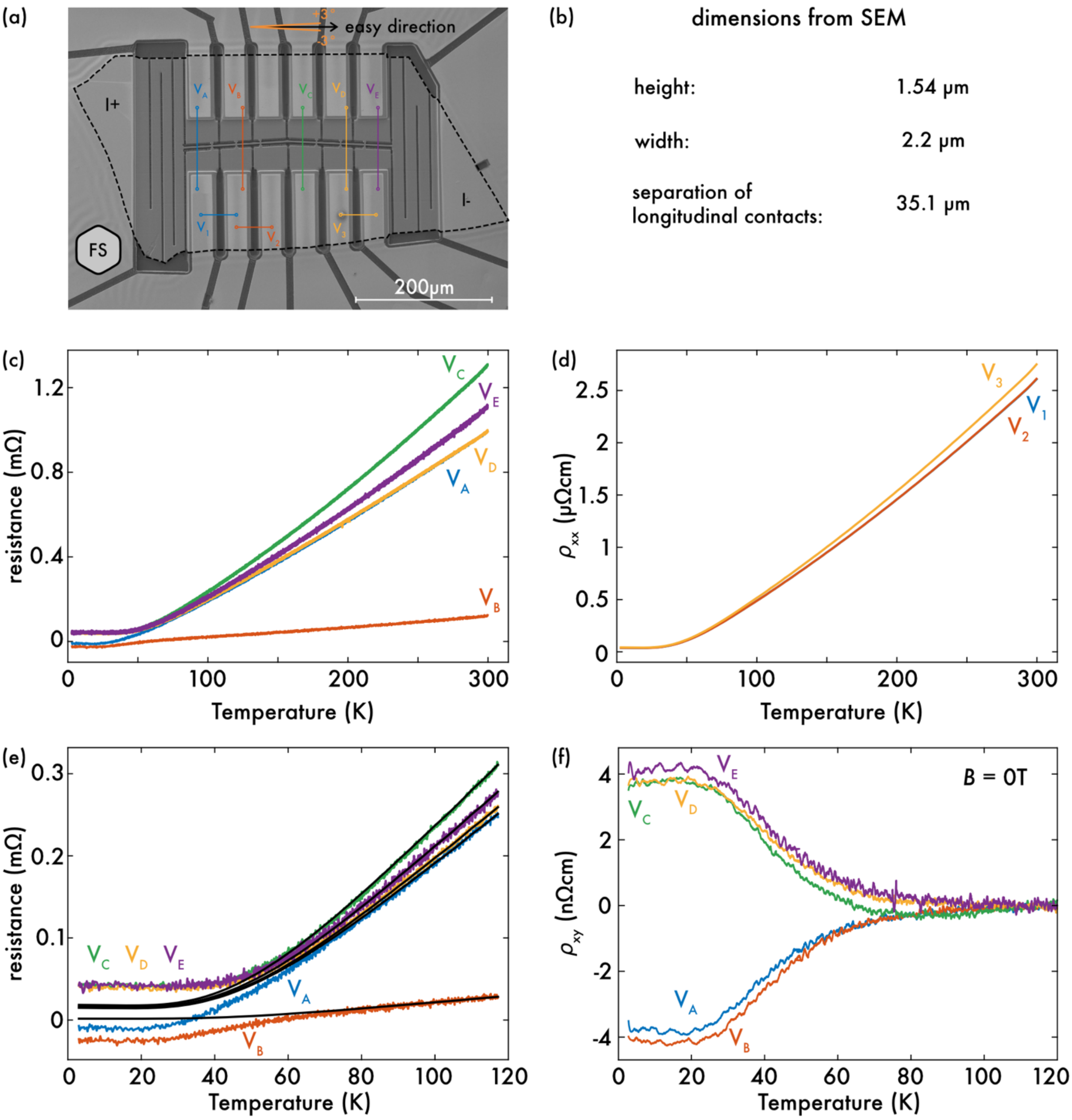

Fig. S9| Raw data of the measurement presented in figure Error! Reference source not found.**4.** (a) SEM image of the FIB-fabricated single crystalline $PdCoO_2$ device. (b) Extracted device dimensions from the SEM images. The error bar on all dimensions is ±150nm. (c) Signal recorded between opposing voltage contacts along the current path. No data could be recorded from the pair between V_B and V_C as a contact broke during the initial cool down of the sample. An excitation current of 500μA was used. (d) Measured longitudinal resistivity between the contacts indicated in panel (a). (e) The colored curves are the same as in panel (c). The black curves are the longitudinal resistance V1 scaled by a constant factor to fit the transverse curves above 120K. The constant factors are V_A: 1/263, V_B: 1/2403, V_C: 1/212, V_D: 1/255, and V_E: 1/237 respectively, implying a transverse contact misalignment of less than 170nm. (f) Transverse resistivity after a longitudinal background has been subtracted, setting ρ_{xy} to zero at 120 K. For consistency the theory predictions in Fig. 4d were also zeroed at 120 K.
